# Sex differences in diagnostic stability in first episode psychosis after 1-year follow-up

**DOI:** 10.1192/j.eurpsy.2024.975

**Published:** 2024-08-27

**Authors:** B. Jiménez-Fernández, A. Toll-Privat, D. Bergé-Baquero, N. V. Motta-Rojas, M. Delgado-Marí, T. Legido-Gil, L. Martínez-Sarduní, T. Legido-Gil, J. Cuevas-Esteban, A. Mané-Santacana

**Affiliations:** ^1^Psychiatry, Hospital Universitari Germans Trias i Pujol, Badalona; ^2^Centro de Investigación Biomédica en Red Salud Mental (CIBERSAM), Madrid; ^3^Institut de Neuropsiquiatria i Addiccions (INAD), Parc Salut Mar; ^4^ Fundació Hospital del Mar Medical Research Institute (IMIM); ^5^Centro de Investigación Biomédica en Red Salud Mental (CIBERSAM), Barcelona, Spain

## Abstract

**Introduction:**

Diagnostic stability is a controversial issue in first episode psychosis (FEP) due to heterogenous symptoms and unclear affective symptoms. Differencing affective and non-affective psychoses is important as treatment strategies are different. Initial affective symptomatology has low specificity for predicting the subsequent diagnosis of affective psychosis. Sex has proven to be relevant for clinical and functional outcomes but it remains unclear how sex may contribute to diagnosis switch of FEP.

**Objectives:**

To determine the role of sex in diagnostic stability in a sample of FEP after 1-year follow-up.

**Methods:**

Diagnoses of FEP patients from Hospital del Mar of Barcelona were assessed at baseline and 1 year after. Univariate analyses was perfomed for all diagnoses and dichotomic variable (affective/non-affective). Logistic regression model was perfomed to know which variables predict diagnosis switch.

**Results:**

256 patients were enrolled. No differences were found at baseline between completers and non-completers (**Table 1).** No significant differences between men and women at baseline diagnosis were found, neither all diagnoses (p=0.274) nor the dichotomic variable affective/non-affective (p=0.829) (**Table 2AB**). Significant differences were found at 1-year follow-up between men and women, for all diagnoses (p=0.043) and the dichotomic variable (p=0.039). Sex was the only variable that predicted diagnosis switch **(Figure 1),** PANSS, CDSS, YMRS, GAF and cannabis did not.

**Table 1.** Baseline characteristics of participants

	Completers (n=188)	Non-completers (n=68)	p
*Women* (n, %)	71 (37.8)	30 (44.1)	0.111
*Age* (M, IQR)	24 (20-28)	22 (20-28)	0.899
*Cannabis use* (M, IQR)	5.5 (0-18)	7 (0-21)	0.231
*DUP* (M, IQR)	45 (12.5-130)	36 (11.25-115.75)	0.213
*PANSS* (m, sd)	44.55 (10.17)	40.93 (10.42)	0.761
*CDSS* (M, IQR)	2 (0-7)	3 (0-5.5)	0.199
*YMRS* (m, sd)	19 (9.64)	17.6 (9.15)	0.845
*GAF* (M, IQR)	30 (25-50)	30 (25-35)	0.114

**TABLE 2A and 2B.** Diagnosis comparison (n, %)

	Baseline	1-year follow-up				
	Men	Women	Total	Men	Women	Total
* Psychosis NOS*	69 (59)	39 (54.9)	108 (57.4)	28 (23.9)	10 (14.1)	38 (20.2)
*Schizophreniform disorder*	22 (18.8)	16 (22.5)	38 (20.2)	14 (12	9 (12.7)	23 (12.2)
*Induced psychosis*	4 (3.4)	0 (0)	4 (2.1)	15 (12.8)	4 (5.6)	19 (10.1)
*Affective psychosis*	17 (14.5)	9 (12.7)	26 (13.8)	24 (20.5)	25 (35.2)	49 (26.1)
*Schizophrenia*	0 (0)	0 (0)	1 (0.4)	30 (25.6)	14 (19.7)	44 (23.4)
*Brief psychotic disorder*	5 (4.3)	7 (9.9)	12 (6.4)	6 (5.1)	8 (11.3)	14 (7.4)

**Table:** 

	Baseline	1-year follow-up				
	Men	Women	Total	Men	Women	Total
* Affective psychosis*	17 (14.5)	9 (12.7)	26 (13.8)	24 (20.5)	25 (35.2)	49 (26.1)
*Non-affective psychosis*	100 (85.5)	62 (87.3)	162 (86.2)	93 (79.5)	46 (64.8)	139 (73.9)

**Image:**

**Figure:**
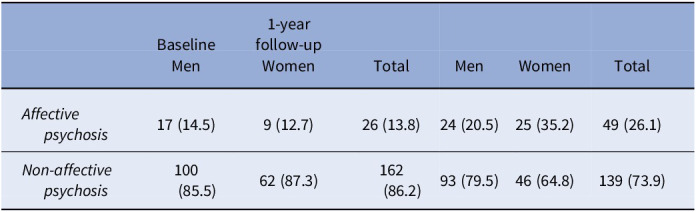

**Conclusions:**

Sex has proven to be the main predictor of switching initial diagnosis of FEP.

**Disclosure of Interest:**

None Declared

